# Barriers to COVID-19 vaccine uptake among resource-limited adults diagnosed with chronic illness

**DOI:** 10.3389/fpubh.2023.1046515

**Published:** 2023-02-09

**Authors:** Lisa P. Spees, Caitlin B. Biddell, Rebekah S. M. Angove, Kathleen D. Gallagher, Eric Anderson, Ashley Christenbury, Gabrielle Rocque, Stephanie B. Wheeler

**Affiliations:** ^1^Department of Health Policy and Management, Gillings School of Global Public Health, University of North Carolina at Chapel Hill, Chapel Hill, NC, United States; ^2^Lineberger Comprehensive Cancer Center, University of North Carolina at Chapel Hill, Chapel Hill, NC, United States; ^3^Patient Advocate Foundation, Hampton, VA, United States; ^4^Division of Hematology and Oncology, Department of Medicine, University of Alabama-Birmingham, Birmingham, AL, United States

**Keywords:** COVID-19, barriers, chronic illness, vaccine hesitancy, vaccine uptake

## Abstract

**Background:**

Despite the use of interventions (e.g., monetary incentives, educational campaigns, on-site workplace vaccination) to increase COVID-19 vaccination, differences in uptake persist by poverty level, insurance status, geography, race, and ethnicity, suggesting that these interventions may not be adequately addressing the barriers faced by these populations. Among a sample of resource-limited individuals with chronic illnesses, we (1) described the prevalence of different types of barriers to the COVID-19 vaccination and (2) identified associations between patients' sociodemographic characteristics and barriers to vaccination.

**Methods:**

We surveyed a national sample of patients with chronic illness and demonstrated healthcare affordability and/or access challenges about barriers to COVID-19 vaccination in July 2021. We categorized participant responses into cost, transportation, informational, and attitudinal barrier domains and assessed the prevalence of each domain, both overall and by self-reported vaccination status. Using logistic regression models, we examined unadjusted and adjusted associations between respondent characteristics (sociodemographic, geographic, and healthcare access) and self-reported barriers to vaccination.

**Results:**

Of 1,342 respondents in the analytic sample, 20% (264/1,342) reported informational barriers and 9% (126/1,342) reported attitudinal barriers to COVID-19 vaccination. Transportation and cost barriers were reported much less commonly, by only 1.1% (15/1,342) and 0.7% (10/1,342) of the sample, respectively. Controlling for all other characteristics, respondents with either a specialist as their usual source of care or no usual source of care had an 8.4 (95% CI: 1.7–15.1) and 18.1 (95% CI: 4.3–32.0) percentage point higher predicted probability, respectively, of reporting informational barriers to care. Compared to females, males had an 8.4% point (95% CI: 5.5–11.4) lower predicted probability of reporting attitudinal barriers. Only attitudinal barriers were associated with COVID-19 vaccine uptake.

**Conclusion:**

Among a sample of adults with chronic illnesses who had received financial assistance and case management services from a national non-profit, informational and attitudinal barriers were more commonly reported than logistical or structural access barriers (i.e., transportation and cost barriers). Interventions should target attitudinal barriers among patients with chronic illness, who may have particular concerns about the interaction of the vaccine with ongoing medical care. Additionally, interventions targeting informational barriers are particularly needed among individuals without a usual source of care.

## Introduction

As of January 2023, more than 30% of individuals in the US have not been fully vaccinated against COVID-19; furthermore, 84% have not received a booster (1). Despite the use of interventions (e.g., monetary incentives, educational campaigns, on-site workplace vaccination) to increase COVID-19 vaccine rates, differences in uptake persist by poverty level, geography, and race in the United States (2). Substantial geographic disparities exist in COVID-19 vaccination uptake at the county-level, with rural areas having 16% lower vaccination than urban areas (3). Similarly, poorer, more disadvantaged counties have a 32% lower vaccination rate than their higher socioeconomic counterparts (4). Despite experiencing higher COVID-19 incidence and mortality due to a host of structural inequities, Black and Hispanic individuals have been consistently shown to be less likely to be vaccinated than White individuals (5–8). These disparities suggest that interventions to increase vaccine uptake may not be adequately addressing the barriers faced by individuals already underserved by the US healthcare system.

Barriers to vaccination may be informational, attitudinal, or structural in nature. Attitudinal barriers, including vaccine hesitancy, have been a major deterrent to COVID-19 vaccination uptake. A scoping review on COVID-19 vaccine hesitancy found that common vaccine-specific factors associated with increased vaccine hesitancy included beliefs that vaccines are not safe/effective and concerns about the rapid development of COVID-19 vaccines (9). Disparate populations, including people of color and those without insurance, often face structural, access-related barriers, further reducing their vaccine uptake (10, 11). For example, despite the execution of Operation Warp Speed, which aimed to make the vaccine widely available to the public at no cost (12), historically marginalized and resource-limited communities continue to face access barriers (8, 11, 13). In a study of over 87,000 participants, Black individuals had significantly lower vaccine uptake, even among those willing to receive the vaccine (8). Qualitative studies have pointed to access-related concerns related to cost and insurance status, language barriers, and the unavailability and logistical complexities of vaccine appointments (11, 13).

Approximately 1 in 6 adults in the US have a chronic health condition (14). Chronic diseases (e.g., cancer, HIV, diabetes, heart disease, hypertension, respiratory disease), by definition, last longer than a year, are functionally debilitating, and require constant monitoring and/or treatment (15). Furthermore, individuals with chronic illnesses that have not have been vaccinated are at heightened risk of experiencing complications, and potential mortality, due to COVID-19 (16). As such, vaccine uptake is a particularly critical preventive measure for this population; in the US, it is estimated that over 32% of individuals with HIV are unvaccinated (17). In other countries, vaccination rates among individuals with cancer, diabetes, and respiratory diseases range from 8 to 39% (18–20).

Several previous studies have described the attitudinal and access barriers to COVID-19 vaccine uptake faced by the general population (11, 21, 22). Outside of the US, individuals with chronic illnesses have pointed to concerns about adverse events, disease decline, and vaccine safety as reasons for vaccine hesitancy (23, 24). In the US, it is critical to determine which types of barriers are faced by individuals with chronic illness to develop targeted and effective interventions (10, 11). In the current study, among a sample of resource-limited individuals with chronic illnesses, we (1) describe the prevalence of different types of barriers to COVID-19 vaccination and (2) identify associations between socio-demographic characteristics and barriers to vaccination.

## Methods

We analyzed cross-sectional survey data from the COVID-19 Impact Survey series collected by Patient Advocate Foundation (PAF) in July 2021. PAF is a national non-profit organization providing financial assistance and case management services to individuals with chronic or life-threatening illnesses, such as cancer, HIV/AIDS, and cardiovascular conditions. PAF emailed the survey to eligible individuals, followed by two reminder emails over the course of 3 weeks.

Eligible individuals had previously received services from PAF (between June 2019 and June 2020) and had opted in to receive survey communications. As PAF provides services to patients living throughout the United States, this sample aims to represent a national sample of patients with limited financial or physical resources. Because this was the third in a series of COVID-19-related surveys, PAF only sent the survey to respondents who completed the first survey, which was administered between May–July 2020. Of the 4,151 individuals who completed the first survey, 1,373 (33%) completed the survey used for this analysis. After excluding 31 individuals (2%) due to missing data for predictor variables included in the multivariable analysis with <10 missing responses, our final analytic sample included 1,342 participants. Supplemental Table 1 includes a comparison of the demographic characteristics between the analytic cohort and the overall cohort of patients served by PAF between June 2019 and June 2020. Compared to the overall cohort of patients served by PAF, the analytic cohort included a higher proportion of individuals who were 36–55 years of age, Non-Hispanic White or Other race, and income ≥$24,000. The University of North Carolina Institutional Review Board deemed this secondary analysis to be non-human subjects research.

### Analyses

Our primary outcome, barriers to COVID-19 vaccination, was measured using the survey question, “Did you have any trouble accessing or receiving the COVID-19 vaccine for any of the following reasons?” which was asked of all participants, regardless of vaccination status. Participants could select multiple response options which we categorized into four domains; specifically, (1) cost barriers included “concerns about cost” and “no insurance,” (2) transportation barriers included “no transportation to get to appointment,” (3) information barriers included “not able to find an available appointment,” “not sure how to make an appointment or where to get vaccinated,” and “not eligible for vaccination,” and (4) attitudinal barriers included “afraid or nervous about the vaccine” and “no time or too busy.” Participants could also select “Other,” which prompted a free-text response. Free-text other responses were coded into these domains by two independent coders (LPS, CBB), and discrepancies were resolved by a third reviewer (SBW). For example, responses such as not wanting to receive the vaccine were coded as attitudinal barriers. Each barrier (i.e., cost, transportation, information, attitudinal) was defined as a binary indicator of whether one or more barriers in each domain was reported.

We assessed the prevalence of each barrier to vaccination, both overall and by self-reported vaccination status (at least one COVID-19 vaccine dose vs. no doses). For all barrier domains with sufficient prevalence in the sample (>10%), we then used logistic regression models to assess unadjusted and adjusted differences between respondent characteristics hypothesized to influence COVID-19 vaccine uptake (25). P-values were calculated using Chi-squared tests or Fisher-Freeman Halton tests [for covariates with more than 2 categories and cell sizes smaller than 5 (26)]. Model fit was assessed based on the Pseudo R^2^ and using the Hosmer and Lemeshow's goodness-of-fit test (27–29). Covariates included in analyses were based on Andersen's model of health care utilization (30, 31) and previous literature examining covariates associated with COVID-19 vaccine uptake acceptance and uptake (6, 32–34). Sociodemographic characteristics included self-reported age, sex, race/ethnicity, and household income. Geographic characteristics included rurality [dichotomized based on the Rural-Urban Commuting Area codes (35): Rural (≥4), Non-rural (<4)] and region of residence. Characteristics related to healthcare access included health insurance coverage and usual source of care. We detected no collinearity in the final models.

Using logistic regression results, we calculated the average marginal effect for each covariate, which can be interpreted as the average difference in the predicted probability of each outcome, holding all other covariates constant, across all observations in the analytic sample. Standard errors and confidence intervals (CIs) for all marginal effects were estimated by applying the Delta method using the “margins” command in STATA 16.1 (StataCorp, College Station, TX).

## Results

Of the 1,342 participants in the analytic sample, the majority were female (58%) and Non-Hispanic White (61%). Though only five percent of participants were between the ages of 19 and 35 years, participant ages were evenly distributed between 36–55 years (31%), 56–65 years (30%), and over 65 years (34%). The majority of respondents were insured by Medicare (64%), had an annual household income less than $48,000 (75%), and reported having a general practitioner as their usual source of care (84%) (Table 1). Overall, 86% of participants self-reported receiving one or more doses of the COVID-19 vaccine, with a higher prevalence among participants over 65 years of age vs. ≤65 (96 vs. 82%) and among Non-Hispanic White participants vs. Black and Hispanic or Latinx (89 vs. 83% vs. 83%) (Supplemental Table 2).

**Table 1:** Descriptive statistics from a sample of patients with chronic or life-threatening illness who received assistance from a national non-profit.

**Sample characteristics**	**Overall** **(*N* = 1,342)**
**Age category**	
19–35 years	68 (5.1%)
36–55 years	415 (30.9%)
56–65 years	408 (30.4%)
>65 years	451 (33.6%)
**Sex**	
Female	777 (57.9%)
Male	565 (42.1%)
**Race/Ethnicity**	
Non-Hispanic White	814 (60.7%)
Non-Hispanic Black	230 (17.1%)
Hispanic or Latinx	72 (5.4%)
Multiple races or Other	139 (10.4%)
Missing	87 (6.5%)
**Usual source of care**	
General practitioner (or LHD)	1121 (83.5%)
Specialist	175 (13.0%)
Other/No usual source	46 (3.4%)
**Annual household income**	
<$24,000	410 (30.6%)
$24,000– <$48,000	598 (44.6%)
$48,000– <$72,000	208 (15.5%)
≧$72,000	108 (8.0%)
Missing	18 (1.3%)
**Health insurance coverage**	
Medicare	854 (63.6%)
Private (ESHI, Marketplace)	322 (24.0%)
Medicaid	113 (8.4%)
Uninsured	28 (2.1%)
Other	25 (1.9%)
**Rurality**	
Non-rural (RUCA<4)	1019 (75.9%)
Rural (RUCA≧4)	323 (24.1%)
**Region**	
South	704 (52.5%)
West	252 (18.8%)
Midwest	192 (14.3%)
Northeast	194 (14.5%)
**Primary diagnosis**	
Cancer	440 (32.8%)
HIV/AIDS	266 (19.8%)
Arthritis/rheumatology disorder	131 (9.8%)
Nervous system and sensory organ disorders	136 (10.1%)
Endocrine, nutritional, metabolic, and immune disorders	99 (7.4%)
Other	270 (20.1%)

**Figure 1:**
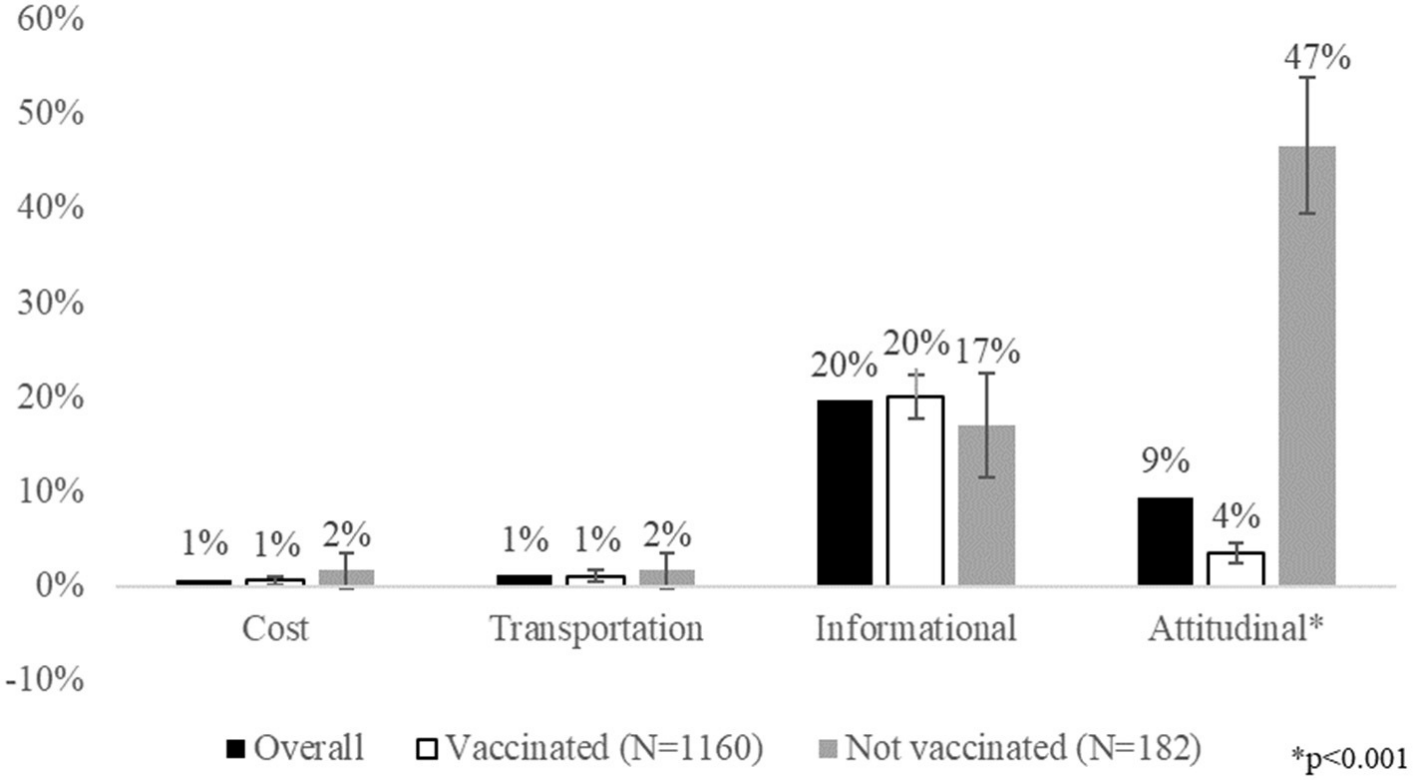
Patient-reported barriers to COVID-19 vaccination by vaccination status (*N* = 1,342). It shows the prevalence of patient-reported barriers to COVID-19 vaccination, both overall and by vaccination status. Cost barriers include concerns about cost of the vaccine itself and not having insurance. Transportation barriers refer to concerns about getting to a vaccine appointment. Informational barriers include not being able to find an available appointment, not being sure how to make an appointment or where to get vaccinated, and questions about eligibility for the vaccine. Attitudinal barriers include being afraid or nervous about the vaccine, not having time to get the vaccine, or not wanting the vaccine. Attitudinal barriers were statistically significantly associated with vaccine uptake (*p < 0.001).

Informational barriers were reported most commonly by 20% (264/1,342) of the sample, followed by attitudinal barriers (9%, 126/1,342). Transportation and cost barriers were reported much less commonly, by only 1.1% (15/1,342) and 0.7% (10/1,342) of the sample, respectively. Only attitudinal barriers were associated with COVID-19 vaccine uptake (*p* < 0.001); compared to only 4% (41/1160) of the vaccinated sample, 47% of unvaccinated respondents (85/182) reported attitudinal barriers (Figure 1).

In unadjusted analysis, only race/ethnicity and usual source of care were associated with reporting informational barriers. Informational barriers were more commonly reported by Non-Hispanic White respondents (24%) compared to Non-Hispanic Black (6%) and Hispanic or Latinx (8%) respondents. Whereas informational barriers were reported by only 18% of respondents with a general practitioner as their usual source of care, informational barriers were reported more commonly by respondents with a specialist as their usual source of care (28%) and respondents with no usual source of care (37%) (Table 2). These differences remained in multivariable analysis. Controlling for all other respondent characteristics, Non-Hispanic Black respondents, compared to Non-Hispanic White respondents, had a 17.7% point (95% CI: 13.3–22.1) lower predicted probability of reporting informational barriers, and Hispanic or Latinx respondents had a 15.0% point (95% CI: 7.7–22.4) lower predicted probability compared to Non-Hispanic Whites. Compared to respondents with a general practitioner as their usual source of care, respondents with a specialist had an 8.4% point (95% CI: 1.7–15.1) higher predicted probability of reporting informational barriers, and respondents without a usual source of care had an 18.1 (95% CI: 4.3–32.0) higher predicted probability (Table 2). Additionally, the Pseudo R^2^ was 0.0585, and the Hosmer and Lemeshow's goodness-of-fit test was insignificant (*p* = 0.4926), indicating good model fit.

**Table 2:** Unadjusted and adjusted associations between socio-demographic characteristics and informational barriers to COVID-19 vaccination (*N* = 1,342).

***N***	**Unadjusted prevalence of informational barriers^a^**	***p* ^b^**	**Adjusted average marginal effects^c^**	**95% Confidence intervals**
	264/1342 (20%)			
**Age category**		0.90		
19–35 years	11/68 (16%)		*ref*	
36–55 years	82/415 (20%)		0.043	−0.064–0.149
56–65 years	82/408 (20%)		0.014	−0.096–0.124
>65 years	89/451 (20%)		0.001	−0.114–0.115
**Sex**		0.56		
Female	157/777 (20%)		*ref*	
Male	107/565 (19%)		−0.025	−0.069–0.019
**Race/Ethnicity**		<0.001		
Non-Hispanic White	196/814 (24%)		*ref*	
Non-Hispanic Black	14/230 (6%)		−0.177^***^	−0.221−0.133
Hispanic or Latinx	6/72 (8%)		−0.150^***^	−0.224−0.077
Multiple races or Other	28/139 (20%)		−0.026	−0.102–0.049
Missing	20/87 (23%)		−0.004	−0.099–0.090
**Usual source of care**		<0.001		
General Practitioner (or LHD)	198/1121 (18%)		*ref*	
Specialist	49/175 (28%)		0.084^**^	0.017–0.151
Other/No usual source	17/46 (37%)		0.181^**^	0.043–0.320
**Annual household income**		0.27		
<$24,000	73/410 (18%)		*ref*	
$24,000– <$48,000	119/598 (20%)		0.015	−0.036–0.065
$48,000– <$72,000	48/208 (23%)		0.034	−0.034–0.101
≧$72,000	18/108 (17%)		−0.036	−0.113–0.041
Missing	6/18 (33%)		0.124	−0.095–0.343
**Health insurance coverage**		0.567^d^		
Medicare	180/854 (21%)		*ref*	
Private (ESHI, Marketplace)	54/322 (17%)		−0.043	−0.099–0.014
Medicaid	21/113 (19%)		−0.02	−0.103–0.063
Other	4/25 (16%)		−0.056	−0.200–0.089
Uninsured	5/28 (18%)		−0.01	−0.168–0.148
**Rurality**		0.81		
Non-rural (RUCA<4)	199/1019 (20%)		*ref*	
Rural (RUCA≧4)	65/323 (20%)		−0.003	−0.051–0.046
**Region**		0.21		
South	125/704 (18%)		*ref*	
West	51/252 (20%)		−0.009	−0.064–0.045
Midwest	41/192 (21%)		0.016	−0.048–0.079
Northeast	47/194 (24%)		0.033	−0.031–0.096

LHD, Local Health Department; ESHI, Employer-sponsored health insurance; RUCA, Rural-Urban Commuting Area.

a Informational barriers include “Not able to find available appointment,” “Not sure how to make appointment or where to get vaccinated,” and “Not eligible for vaccine.”

b *p*-values calculated using Chi-squared tests unless otherwise noted.

c Multivariable logistic regression (Pseudo R^2^ = 0.0585) used to estimate average marginal effects (standard errors reported in parentheses). Average marginal effects represent the average difference in the predicted probability of reporting facing informational barriers to COVID-19 vaccination holding all other covariates constant, across all observations in the analytic sample.

d *p*-value calculated using the Fisher-Freeman-Halton test due to cell sizes <5.

*** *p* < 0.01,

** *p* < 0.05, ^*^p < 0.1.

In unadjusted analysis, attitudinal barriers were associated with age, sex, race/ethnicity, and health insurance coverage (Table 3). However, after controlling for all sociodemographic and healthcare access characteristics, most associations were attenuated. Compared to females, males had an 8.4% point (95% CI: 5.5–11.4) lower predicted probability of reporting attitudinal barriers. Additionally, respondents reporting multiple races or other race/ethnicity had a 6.6% point (95% CI: 0.6–12.5) higher predicted probability of reporting attitudinal barriers compared to Non-Hispanic White respondents. Though only borderline significant, respondents over 65 years of age (compared to respondents between 19–35 years of age) had a 7.0% point (95% CI: 1.3–15.3) lower predicted probability of reporting attitudinal barriers, and uninsured respondents (compared to Medicare-insured) had a 10.9% point (95% CI: −1.9–23.7) higher predicted probability of reporting attitudinal barriers (Table 3). For this multivariable analysis, the Pseudo R^2^ was 0.1083, and the Hosmer and Lemeshow's goodness-of-fit test was insignificant (*p* = 0.338), indicating good model fit.

**Table 3:** Unadjusted and adjusted associations between socio-demographic characteristics and attitudinal barriers to COVID-19 vaccination (*N* = 1,342).

***N***	**Unadjusted Prevalence of Attitudinal Barriers^a^**	***p* ^b^**	**Adjusted Average Marginal Effects^c^**	**95% Confidence Intervals**
	126/1342 (9%)			
**Age category**		<0.001		
19–35 years	12/68 (18%)		*ref*	
36–55 years	61/415 (15%)		−0.01	−0.087–0.067
56–65 years	34/408 (8%)		−0.045	−0.125–0.035
>65 years	19/451 (4%)		−0.070^*^	−0.153–0.013
**Sex**		<0.001		
Female	108/777 (14%)		*ref*	
Male	18/565 (3%)		−0.084^***^	−0.114–−0.055
**Race/Ethnicity**		0.002		
Non-Hispanic White	55/814 (7%)		*ref*	
Non-Hispanic Black	31/230 (14%)		0.026	−0.015–0.067
Hispanic or Latinx	8/72 (11%)		0.021	−0.046–0.089
Multiple races or Other	20/139 (14%)		0.066^**^	0.006–0.125
Missing	12/87 (14%)		0.056	−0.015–0.126
**Usual source of care**		0.061		
General Practitioner (or LHD)	97/1121 (9%)		*ref*	
Specialist	21/175 (12%)		0.032	−0.017–0.081
Other/No usual source	8/46 (17%)		0.058	−0.035–0.151
**Annual household income**		0.029^d^		
<$24,000	48/410 (12%)		*ref*	
$24,000– <$48,000	43/598 (7%)		−0.021	−0.058–0.015
$48,000– <$72,000	17/208 (8%)		−0.004	−0.056–0.047
≧$72,000	15/108 (14%)		0.021	−0.045–0.087
Missing	3/18 (17%)		−0.008	−0.116–0.099
**Health insurance coverage**		<0.001^d^		
Medicare	61/854 (7%)		*ref*	
Private (ESHI, Marketplace)	34/322 (11%)		−0.015	−0.053–0.023
Medicaid	20/113 (18%)		0.025	−0.033–0.083
Other	2/25 (8%)		−0.035	−0.117–0.047
Uninsured	9/28 (32%)		0.109^*^	−0.019–0.237
**Rurality**		0.71		
Non-rural (RUCA<4)	94/1019 (9%)		*ref*	
Rural (RUCA≧4)	32/323 (10%)		0.008	−0.029–0.044
**Region**		0.46		
South	73/704 (10%)		*ref*	
West	22/252 (9%)		−0.011	−0.054–0.033
Midwest	18/192 (9%)		−0.006	−0.053–0.041
Northeast	13/194 (7%)		−0.032	−0.075–0.010

LHD, Local Health Department; ESHI, Employer-sponsored health insurance; RUCA, Rural-Urban Commuting Area.

a Attitudinal barriers include “Afraid or nervous about the vaccine,” “No time or too busy,” and Didn't want the vaccine (written in).

b *p*-values calculated using Chi-squared tests unless otherwise noted.

c Multivariable logistic regression (Pseudo R^2^ = 0.1083) used to estimate average marginal effects (standard errors reported in parentheses). Average marginal effects represent the average difference in the predicted probability of reporting facing attitudinal barriers to COVID-19 vaccination holding all other covariates constant, across all observations in the analytic sample.

d *p*-value calculated using the Fisher-Freeman-Halton test due to cell sizes < 5.

*** *p* < 0.01,

** *p* < 0.05,

* *p* < 0.1.

In sensitivity analyses, we explored the effect of adding Covid-19 vaccine uptake as a covariate in the multivariable models for both informational and attitudinal barriers. On the whole, adding vaccination status did not change the results of the associations between patient characteristics and vaccine barriers. As shown in Figure 1 as well, vaccination status was statistically significantly associated with reporting attitudinal barriers but not informational barriers (Supplemental Table 3).

## Discussion

Among a sample of resource-limited adults with chronic illnesses, informational and attitudinal barriers were more commonly reported than structural access barriers (i.e., transportation and cost barriers). Attitudinal barriers were associated with lower vaccine uptake. In particular, attitudinal barriers were more prevalent among females (compared to males) and uninsured (compared to insured individuals). Interestingly, Black and Latinx or Hispanic individuals were less likely to face informational barriers than their White counterparts. Participants with either a specialist as their usual source of care or no usual source of care were more likely to report informational barriers compared to those whose usual source of care was a general practitioner. As ongoing COVID-19 vaccination continues to be a primary defense against the pandemic, our findings aim to inform efforts to develop interventions that are needed to overcome vaccine hesitancy and improve access to vaccine uptake in the national response to COVID-19 (36).

Even prior to the COVID-19 pandemic, having a usual source of care has been considered essential to improving or maintaining individual's health, especially among individuals with chronic illness. Having a usual source of care facilitates timely receipt of preventative services, including immunizations (37–42), as well as fewer emergency department visits and hospital admissions (43). Among a nationally representative population, older, adult men (ages 50–64) and women (ages 40–64) with a usual source of care had, respectively, almost 10 times higher odds of receiving a Prostate Specific Antigen test and 5 times higher odds of receiving a mammogram compared with to those without a usual source of care (44). During the COVID-19 pandemic, having a usual source of care facilitated access to COVID-19 testing and treatment services (45). For example, primary care providers could directly contact patients to encourage them to vaccinate; a randomized controlled trial found that outreach *via* electronic secure messages and mailings from an individual's regular primary care physician increased COVID-19 vaccination rates among older Black and Latinx/Hispanic adults (46).

The rate of COVID-19 vaccination is significantly lower among Black and Hispanic or Latinx populations (5–8). Several studies suggest that this is due to vaccine hesitancy among these historically marginalized populations (32, 47, 48). Even in our study, the proportion of individuals of color reporting attitudinal barriers was higher than those reporting informational barriers; in fact, the percent Black participants reporting attitudinal barriers was more than twice the percent reporting informational barriers. While we found that Black and Hispanic/Latinx respondents were significantly less likely to report informational barriers than White respondents, this is potentially because these individuals have experienced systemic racism and consequently are less likely to receive the vaccine or to seek out information on the vaccine; in other words, they avoided facing informational barriers altogether due to mistrust of healthcare systems. Indeed, even among individuals with chronic conditions that need regular medical care, Black individuals have demonstrated high vaccine hesitancy (49). Alternatively, however, this could also be due to the success of outreach programs that specifically targeted Black and Latinx/Hispanic individuals; programs focused on equity-based vaccine allocation and community engagement have been shown to almost double vaccination rates (50). Furthermore, a recent study found that, over the course of the pandemic, the rate of vaccine hesitancy has been decreasing more rapidly among Black individuals than White individuals (29). Future research on strategies to increase COVID-19 vaccination among people of color should continue to explore the barriers faced be these populations and develop tailored, and potentially multicomponent, interventions to address the identified barriers.

About half as many uninsured U.S. adults have received the COVID-19 vaccine as insured adults (51). The U.S. Department of Health and Human Services established the COVID-19 Uninsured Program, which reimburses providers at national Medicare rates, to help uninsured individuals access COVID-19 testing, vaccinations, and treatment (52). While this program addresses access barriers, our results suggest uninsured patients are more likely to face attitudinal barriers to COVID-19 uptake. Consequently, addressing vaccine hesitancy, in addition to addressing access barriers, is needed to improve vaccine uptake among the uninsured population.

While females are more likely to practice preventative behaviors such as wearing face masks to prevent COVID-19 infections (53, 54), COVID-19 vaccine refusal is significantly higher among females than males in the U.S (33, 47, 48). A large national study of adult Americans found the odds of expressing vaccine hesitancy were 44% higher in females than males (53). Even among adults with chronic diseases, sex differences persist. A study among immunocompromised individuals found that females were more likely to express vaccine hesitancy than males (55, 56); in particular, females expressed concerns about the safety of vaccinations and were more often worried about vaccine side effects (55). With the approval of the COVID-19 vaccine for children, these results are particularly troubling, as women are more often responsible for making medical decisions for their children than men (57). These differences in COVID-19 attitudes by sex, which we also found in this study, suggest that sex- and gender-specific concerns should be addressed and targeted when educating females about vaccinations or designing vaccination campaigns.

Several limitations of this study should be noted. First, results from this study may not be generalizable to the full US population of adults with chronic illness due to the relatively low response rate of 33% and that the sample was drawn from a surveyed population who sought financial assistance or social need navigation from a national non-profit. Given the resource constraints faced by these individuals, it is possible that our estimates may be biased as these individuals may have been more susceptible to experiencing vaccine-related barriers. In contrast, their prior experience accessing resources from a national non-profit may suggest a lower likelihood of facing access barriers to vaccination. Furthermore, while our analysis included several different types of barriers, we did not explore how these barriers may have interacted or influenced one another; for example, individuals may have not even tried to make a vaccine appointment (i.e., they altogether avoided facing informational barriers) if they believed they could not afford the vaccine or have the necessary transportation to attend a vaccine clinic appointment. Future qualitative research should further explore the nuances of the relationships between different types of barriers.

At the same time, our study has several strengths. First, because PAF is a nationally-recognized organization, the study sample includes individuals from across the US. Second, in contrast to previous studies that focused on describing barriers to vaccination, the present study quantitatively expresses different types of barriers. Finally, while the majority of the literature on barriers to COVID-19 vaccination have focused on the general population, this study is the first to examine barriers among those with chronic illness in the US.

## Conclusion

Over 80% of the US population has received at least one dose of a COVID-19 vaccine (1). However, several rounds of booster vaccination shots have been recommended, depending on eligibility, and future booster vaccines are currently being developed. While a multitude of interventions and programs have been developed to improve vaccine uptake, in order to increase both effectiveness and efficiency of vaccine uptake, interventions should proactively tailor strategies to the most prevalent barriers encounters by target populations. In particular, our results among resource-limited individuals with chronic illness, campaigns providing information on finding and scheduling available vaccine appointments are needed for those without a usual source of care. Programs to address attitudinal barriers, particularly concerns about vaccine safety, should be targeted toward females and uninsured individuals.

## Data availability statement

The datasets presented in this article are not readily available because this data is owned by Patient Advocate Foundation and is subject to a Data Use Agreement. Requests to access the datasets should be directed to Patient Advocate Foundation, https://www.patientadvocate.org/.

## Author contributions

LS: conceptualization, methodology, formal analysis, and writing—original draft preparation. CB: conceptualization, methodology, writing—original draft preparation, and review and editing. RA and KG: investigation, conceptualization, and writing—review and editing. EA: data curation, conceptualization, and writing—review and editing. GR: writing—review and editing. SW: conceptualization, methodology, supervision, and writing—review and editing. All authors contributed to the article and approved the submitted version.
