# Supplementary material for: Barriers to COVID-19 vaccine uptake among resource-limited adults diagnosed with chronic illness
Source: Front Public Health. 2023 Feb 9;11:1046515. doi: 10.3389/fpubh.2023.1046515 (PMC9948023; doi:10.3389/fpubh.2023.1046515)
Supplement: Supplementary file 1 [file Table_1.DOCX]

**Supplemental Appendix**

**Supplemental Table 1.** Comparison of sociodemographic characteristics between analytic cohort and eligible patients served by the Patient Advocate Foundation between June 2019 and June 2020

|  | **Patients Served** | **Analytic Cohort** |
| --- | --- | --- |
| **N** | **N= 103,526** | **N = 1342** |
| **Age Category** |  |  |
| ≤18 years | 1,044 (1%) | 0 (0%) |
| 19 - 35 years | 6,723 (6%) | 68 (5%) |
| 36 - 55 years | 20,174 (20%) | 415 (31%) |
| ≥56 years | 73,568 (71%) | 859 (64%) |
| Missing | 2,017 (2%) | 0 (0%) |
| **Sex** |  |  |
| Female | 58,169 (56%) | 777 (58%) |
| Male | 44,752 (43%) | 565 (42%) |
| Missing | 605 (1%) | 0 (0%) |
| **Race/Ethnicity** |  |  |
| Non-Hispanic White | 45,148 (44%) | 814 (61%) |
| Non-Hispanic Black | 17,794 (17%) | 230 (17%) |
| Hispanic or Latinx | 7,051 (7%) | 72 (5%) |
| Multiple races or Other | 4,785 (5%) | 139 (10%) |
| Missing | 28,748 (28%) | 87 (7%) |
| **Annual household income** |  |  |
| <$24,000 | 39,241 (38%) | 410 (31%) |
| $24,000 - <$48,000 | 40,490 (39%) | 598 (45%) |
| $48,000 - <$72,000 | 13,629 (13%) | 208 (15%) |
| >=$72,000 | 4,591 (4%) | 108 (8%) |
| Missing | 5,575 (5%) | 18 (1%) |
| **Region** |  |  |
| South | 52,128 (50%) | 704 (53%) |
| West | 15,845 (15%) | 252 (19%) |
| Midwest | 19,051 (18%) | 192 (14%) |
| Northeast | 14,277 (14%) | 194 (14%) |
| Missing | 2,225 (2%) | 0 (0%) |

**Supplemental Table 2.** Sociodemographic characteristics by vaccine uptake

|  | **Not vaccinated** | **Fully or partially vaccinated** | **p-value** |
| --- | --- | --- | --- |
| **N** | **182** | **1160** |  |
| **Age Category** |  |  | <0.001 |
| 19 - 35 years | 22 (12.1%) | 46 (4.0%) |  |
| 36 - 55 years | 100 (54.9%) | 315 (27.2%) |  |
| 56 - 65 years | 41 (22.5%) | 367 (31.6%) |  |
| >65 years | 19 (10.4%) | 432 (37.2%) |  |
| **Sex** |  |  | <0.001 |
| Female | 149 (81.9%) | 628 (54.1%) |  |
| Male | 33 (18.1%) | 532 (45.9%) |  |
| **Race/Ethnicity** |  |  | 0.037 |
| Non-Hispanic White | 91 (50.0%) | 723 (62.3%) |  |
| Non-Hispanic Black | 40 (22.0%) | 190 (16.4%) |  |
| Hispanic or Latinx | 12 (6.6%) | 60 (5.2%) |  |
| Multiple races or Other | 23 (12.6%) | 116 (10.0%) |  |
| Missing | 16 (8.8%) | 71 (6.1%) |  |
| **Usual source of care** |  |  | <0.001 |
| General Practitioner (or LHD) | 136 (74.7%) | 985 (84.9%) |  |
| Specialist | 33 (18.1%) | 142 (12.2%) |  |
| Other/No usual source | 13 (7.1%) | 33 (2.8%) |  |
| **Annual household income** |  |  | 0.17 |
| <$24,000 | 64 (35.2%) | 346 (29.8%) |  |
| $24,000 - <$48,000 | 67 (36.8%) | 531 (45.8%) |  |
| $48,000 - <$72,000 | 29 (15.9%) | 179 (15.4%) |  |
| >=$72,000 | 18 (9.9%) | 90 (7.8%) |  |
| Missing | 4 (2.2%) | 14 (1.2%) |  |
| **Health insurance coverage** |  |  | <0.001 |
| Medicare | 81 (44.5%) | 773 (66.6%) |  |
| Private (ESHI, Marketplace) | 49 (26.9%) | 273 (23.5%) |  |
| Medicaid | 36 (19.8%) | 77 (6.6%) |  |
| Uninsured | 7 (3.8%) | 18 (1.6%) |  |
| Other | 9 (4.9%) | 19 (1.6%) |  |
| **Rurality** |  |  | 0.037 |
| Non-rural (RUCA<4) | 127 (69.8%) | 892 (76.9%) |  |
| Rural (RUCA>=4) | 55 (30.2%) | 268 (23.1%) |  |
| **Region** |  |  | 0.023 |
| South | 112 (61.5%) | 592 (51.0%) |  |
| West | 32 (17.6%) | 220 (19.0%) |  |
| Midwest | 23 (12.6%) | 169 (14.6%) |  |
| Northeast | 15 (8.2%) | 179 (15.4%) |  |
| **Primary Diagnosis** |  |  | <0.001 |
| Cancer | 64 (35.2%) | 376 (32.4%) |  |
| HIV/AIDS | 12 (6.6%) | 254 (21.9%) |  |
| Arthritis/Rheumatology Disorder | 29 (15.9%) | 102 (8.8%) |  |
| Nervous system and sensory organ  disorders | 28 (15.4%) | 108 (9.3%) |  |
| Endocrine, nutritional, metabolic,  and immune disorders | 17 (9.3%) | 82 (7.1%) |  |
| Other | 32 (17.6%) | 238 (20.5%) |  |

Abbreviations: LHD (Local Health Department); ESHI (Employer-sponsored health insurance); RUCA (Rural-Urban Commuting Area)

P-values calculated using Chi-squared test or Fisher's exact test if cell size less than 5.

**Supplemental Table 3.** Adjusted associations between socio-demographic characteristics and barriers to COVID-19 vaccination, controlling for COVID-19 vaccine uptake (N=1342)

|  | Informational Barriers | | Attitudinal Barriers | |
| --- | --- | --- | --- | --- |
|  | Average Marginal Effects | 95% Confidence Intervals | Average Marginal Effects | 95% Confidence Intervals |
| **Age category** *(ref: 19 – 35 years)* |  |  |  |  |
| 36 - 55 years | 0.040 | -0.069 - 0.149 | 0.008 | -0.039 - 0.056 |
| 56 - 65 years | 0.006 | -0.107 - 0.119 | 0.021 | -0.033 - 0.075 |
| >65 years | -0.008 | -0.126 - 0.109 | 0.010 | -0.050 - 0.070 |
| **Sex** *(ref: Female)* |  |  |  |  |
| Male | -0.028 | -0.073 - 0.016 | -0.060*** | -0.088 - -0.031 |
| **Race/Ethnicity** *(ref: Non-Hispanic White)* | |  |  |  |
| Non-Hispanic Black | -0.177*** | -0.221 - -0.133 | 0.032* | -0.006 - 0.070 |
| Hispanic or Latinx | -0.150*** | -0.223 - -0.076 | 0.020 | -0.038 - 0.078 |
| Multiple races or Other | -0.025 | -0.101 - 0.051 | 0.051** | 0.002 - 0.099 |
| Missing | -0.001 | -0.096 - 0.094 | 0.037 | -0.019 - 0.093 |
| **Usual source of care** *(ref: General practitioner or LHD)* | | |  |  |
| Specialist | 0.086** | 0.019 - 0.153 | 0.009 | -0.030 - 0.048 |
| Other/No usual source | 0.189*** | 0.049 - 0.329 | 0.002 | -0.058 - 0.061 |
| **Annual household income** *(ref: <$24,000)* | |  |  |  |
| $24,000 - <$48,000 | 0.015 | -0.035 - 0.065 | -0.021 | -0.054 - 0.012 |
| $48,000 - <$72,000 | 0.035 | -0.032 - 0.103 | -0.016 | -0.058 - 0.027 |
| >=$72,000 | -0.035 | -0.112 - 0.043 | 0.006 | -0.049 - 0.060 |
| Missing | 0.120 | -0.099 - 0.338 | 0.011 | -0.105 - 0.127 |
| **Health insurance coverage** *(ref: Medicare)* | |  |  |  |
| Private (ESHI, Marketplace) | -0.045 | -0.101 - 0.012 | 0.004 | -0.032 - 0.041 |
| Medicaid | -0.014 | -0.099 - 0.071 | -0.007 | -0.048 - 0.035 |
| Other | -0.049 | -0.198 - 0.100 | -0.046 | -0.108 - 0.017 |
| Uninsured | -0.005 | -0.165 - 0.156 | 0.089 | -0.021 - 0.199 |
| **Rurality** *(ref: Non-rural)* |  |  |  |  |
| Rural (RUCA>=4) | -0.001 | -0.050 - 0.047 | -0.010 | -0.040 - 0.020 |
| **Region** *(ref: South)* |  |  |  |  |
| West | -0.010 | -0.065 - 0.044 | 0.009 | -0.029 - 0.046 |
| Midwest | 0.013 | -0.050 - 0.076 | 0.023 | -0.020 - 0.065 |
| Northeast | 0.028 | -0.035 - 0.092 | 0.006 | -0.038 - 0.049 |
| **Covid-19 Vaccine Uptake** *(ref: No vaccines)* | |  |  |  |
| Fully or partially vaccinated | 0.041 | -0.018 - 0.101 | -0.365*** | -0.445 - -0.285 |
| Abbreviations: LHD (Local Health Department); ESHI (Employer-sponsored health insurance); RUCA (Rural-Urban Commuting Area) | | | | |
| *** p<0.01, ** p<0.05, * p<0.1 |  |  |  |  |
